# Genome-scale study of the importance of binding site context for transcription factor binding and gene regulation

**DOI:** 10.1186/1471-2105-9-484

**Published:** 2008-11-17

**Authors:** Jakub Orzechowski Westholm, Feifei Xu, Hans Ronne, Jan Komorowski

**Affiliations:** 1 The Linnaeus Centre for Bioinformatics, Uppsala University, BMC, Box 598, SE-751 24 Uppsala, Sweden; 2 Department of Medical Biochemistry and Microbiology, Uppsala University, BMC, Box 582, SE-751 23 Uppsala, Sweden; 3 Interdisciplinary Centre for Mathematical and Computational Modelling, Warsaw University, 02-106, Warsaw, Poland

## Abstract

**Background:**

The rate of mRNA transcription is controlled by transcription factors that bind to specific DNA motifs in promoter regions upstream of protein coding genes. Recent results indicate that not only the presence of a motif but also motif context (for example the orientation of a motif or its location relative to the coding sequence) is important for gene regulation.

**Results:**

In this study we present ContextFinder, a tool that is specifically aimed at identifying cases where motif context is likely to affect gene regulation. We used ContextFinder to examine the role of motif context in *S. cerevisiae *both for DNA binding by transcription factors and for effects on gene expression. For DNA binding we found significant patterns of motif location bias, whereas motif orientations did not seem to matter. Motif context appears to affect gene expression even more than it affects DNA binding, as biases in both motif location and orientation were more frequent in promoters of co-expressed genes. We validated our results against data on nucleosome positioning, and found a negative correlation between preferred motif locations and nucleosome occupancy.

**Conclusion:**

We conclude that the requirement for stable binding of transcription factors to DNA and their subsequent function in gene regulation can impose constraints on motif context.

## Background

Regulation of gene expression enables cells to respond to external signals such as nutrient availability, stress and signalling molecules, and also allows cells in multicellular organisms to differentiate into different cell types. Gene expression is regulated on many different levels such as chromatin structure, splicing of RNA and post-translational protein modifications, but the most important regulatory step takes place at the level of transcription. The rate of transcription is controlled by transcription factors (TFs) that bind to specific DNA sequences (called *motifs *in the following) in promoter regions upstream of the transcribed sequences. TFs bound to their designated DNA sites can regulate transcription by interacting with the basal transcription machinery or with co-factors, by modifying chromatin structure or by blocking or facilitating access to the DNA for other TFs. The motifs bound by TFs are thus important components in the regulation of gene expression, as they determine which genes different TFs will regulate. Binding sites for many TFs have been characterized [1,2] and several computational approaches have been developed to identify conserved DNA motifs in promoters of co-regulated genes [3-9]. However, the mere presence of a TF-binding motif in a promoter is not sufficient to guarantee it is bound by this TF *in vivo*. In fact, most TF-binding motifs found in promoters have no documented effects on gene expression.

An additional level of complexity comes from the presence of multiple distinct motifs in the same promoter. This can increase the number of possible gene expression patterns, and enables cells to fine-tune the response to different conditions. Moreover, since different TFs can modulate each other's DNA binding and/or activity, the location of different motifs with respect to each other (the promoter context) is also important. Several previous studies [3,10-17] have examined the combinatorial aspects of gene regulation. However, interest has recently focused on the importance of motif context, *i.e. *how geometric constraints such as the location or orientation of a motif can affect gene expression. Genome-wide localization studies have shown patterns of localization of TFs to motifs closely upstream of transcription start sites [18]. When the overall distribution of motifs in promoters bound by TFs was plotted, enrichment within a region a few hundred bp upstream of the start codon was found [19]. Some *de novo *motif finding tools (e.g. [4,20]) used conservation of location as a selection criterion when searching for novel motifs in promoters of co-regulated genes. In an effort to predict gene expression patterns from promoter sequence in yeast [12], motif context in the form of location and orientation was also included in the model. Regulation was modelled separately for groups of co-expressed genes ("regulons"). However, a later study [21] showed that including motif context into the models did not improve predictions of gene expression. Another study [22] modelled the influence of motifs in different contexts on yeast gene expression, without partitioning genes into different sets. Both [12] and [22] took into account the orientations of the motifs and their locations relative to the start codons. The model used in [12] also included combinations of motifs. In [23] the location of motifs was analyzed on a global scale, in promoters of genes sharing functional annotations in human and mouse. One study [17] also examined the importance of motif context for combinatorial gene regulation, by studying distances between pairs of motifs. A recent study [24] presented a motif finding approach where the discovered motifs were further characterized in terms of location and orientation bias. However, none of the above studies has carried out an examination on a global scale where patterns of motif location and orientation relative the coding sequence were correlated with TF-DNA interactions and as well as with gene expression.

In addition to factors such as the locations and orientations of TF-binding motifs, nucleosome occupancy in promoters is also an important predictor of the biological effects of these sites. In most cases, nucleosomes inhibit transcription by blocking access to DNA so that TFs and the basal transcription machinery cannot bind. Consistent with this, promoters of highly transcribed genes are usually depleted of nucleosomes as compared to genes with lower expression [13,25,26]. Moreover, active TF binding sites that are bound by TFs are usually depleted of nucleosomes as compared to inactive (cryptic) sites [27,28].

Except for one study [23], the studies mentioned above were carried out in yeast. The yeast *S. cerevisiae *has been the organism of choice when studying regulation of gene expression in eukaryotes. There are several reasons for this, such as the availability of genome wide data on mRNA transcription (for example [29-31]) and TF-DNA interactions [19,32], the availability of knockout mutants for all yeast genes, including all TFs, and the fact that yeast has a compact genome with small and well-defined promoters.

In this study we have carried out a genome-scale examination of the importance of motif context for both TF-DNA interactions and gene expression in *S. cerevisiae*. This was done using ContextFinder, a new tool we have designed to identify cases where motif context is likely to be important for gene regulation. For the purpose of this study, we define motif context as the location and the orientation of the motif relative to the start codon, since the distance between transcription start site and start codon is usually fixed in yeast [33-35], and since the position of the start codon always is known (see Methods). It is worth pointing out that the problem investigated in this study is a different problem than the one discussed in previous studies [12,22], where the aim was to model gene expression, and information about motif context was included in the models. Here, instead of modelling gene expression, we are interested in finding and characterizing cases where motif location and orientation appears to be important for gene regulation, irrespective of the details of this regulation. Our approach is thus related to those used in [23] and [24]. However, our study differs in two aspects. The first aspect is the data. Tabach et al. [23] primarily used groups of genes sharing a functional annotation to approximate co-regulation, and also investigated the effects of the locations of six specific motifs on gene expression. The study by Elemento et al. [24] examined the orientation and location of 23 yeast motifs in connection with gene expression data. In contrast, we have examined 150 yeast motifs both in co-expressed promoters and in promoters bound by the same TF. The data used in our study covers a wider range of yeast motifs and is closely connected to the biological function of the motifs in terms of both TF binding and gene regulation. Consequently, basing analysis on these data is likely to provide a more accurate picture of the effects of motif context. The other new aspect in our work is methodological: The method used in [23] was based on performing separate tests for motif enrichment within different regions of the promoter. This results in many p-values (one for each region), without any obvious statistical interpretation with regard to the overall bias in motif location. Moreover, that study did not consider motif orientation. The method used in [24] used a randomization test to provide a single p-value for location bias. However, no significance measure was provided for the orientation bias. Instead, orientation bias was reported if one orientation of a motif contained significant information about gene expression (compared to a threshold) but not the other orientation. A drawback of that approach is that the two orientations are not compared directly to each other, but only to the significance threshold. In contrast the method presented here fits a model to the motif distribution and specifically looks for differences in orientation and location between a set of active promoters and a background set of promoters. Two p-values are returned, one for bias in location and one for bias in orientation, making the results easy to interpret.

## Results

We have developed a method, implemented in a program called ContextFinder that can identify cases where motif context is likely to be important for gene regulation. The basic idea behind ContextFinder is to look for differences between a selected set of promoters (for example promoters bound by a given TF or promoters of co-expressed genes) and a control set (typically all other promoters except the selected set). The differences of interest to us are the locations and orientations of a specific motif. This tool is then used together with experimental data to study how common location and orientation bias is, for DNA binding and for regulation of gene expression.

### Data and Procedure

ContextFinder takes as input a selected set of promoters, a control set and a motif. The underlying assumption is that motifs found in the selected set are biologically active in some way (for instance, by binding TFs and/or regulating gene expression) while motifs in the control set are not. We proceed to determine if the distribution of motifs in the selected set of promoters is significantly different from the control set. This is done by fitting a model to the data in which the motif frequency depends on the set that the promoter belongs to, the location within the promoter, the orientation of the motif and interactions between these factors. Significance in the form of p-values for *location bias *(difference in location between the selected set and the control set) and *orientation bias *(difference in orientation between the two sets) are then computed from the model. For a detailed description of the procedure, see the Methods section. A web interface to ContextFinder is available at [36].

To carry out an genome-wide study of motif context that goes beyond looking at a few individual examples, we used a comprehensive list of known yeast motifs [37] together with sets of genes derived from data describing DNA binding of TFs [19,32] and gene co-expression data [12]. All motifs were tested against all sets of genes in order to identify cases where a known motif is enriched in a given set of promoters. ContextFinder was then applied to all such cases (in total, 280 for the TF binding data and 23 for the gene expression data). We focused our studies on protein encoding genes for two reasons. Firstly, the vast majority of all TFs are involved in regulating such genes, which accounts for much of the complexity in gene expression. Secondly, it is easy to define the location of a motif by using the start of the open reading frame as a point of reference, even if the transcription start site has not been mapped for a given gene.

### Motif location is important for DNA binding of transcription factors

The TF-DNA interaction data from [19,32] contains data from 350 experiments on DNA binding of different TFs under different conditions. We first used this set of data to study if there is a bias in the location and/or orientation of motifs within promoters known to bind a given TF, as compared to other promoters containing the same motif. Our results (first row in Table 1) suggest that the location of motifs within promoters is important for DNA binding, as location bias was found in 40% (113) of 280 motif-experiment pairs tested. In contrast, motif orientation does not appear to be crucial for DNA binding, as orientation bias was not found in any of the pairs.

**Table 1:** Frequencies of location and orientation bias in motif-experiment pairs

**Data**	**nr of pairs examined**	**location bias**	**orientation bias**	**any bias**
DNA binding	280	113 (40%)	0 (0%)	113 (40%)
DNA binding (only unique promoters)	105	42 (40%)	2 (2%)	42 (40%)
co-expression	23	15 (65%)	5 (22%)	15 (65%)

Cases of divergently transcribed genes, where the DNA binding data from the shared promoter region is mapped to both genes, are a potential problem. In such cases a certain motif may be important only for regulation of one of the two genes, and it is its position with respect to the coding region of that gene that matters. The contribution from the other gene will obscure patterns of location and orientation bias. To avoid this problem, we also performed the analysis on a subset of the DNA binding data, where only promoters that were mapped to a single gene were considered. The results are shown in the second row of Table 1. Although fewer motif-experiment instances were examined in this case, the overall results were similar.

**Figure 1:**
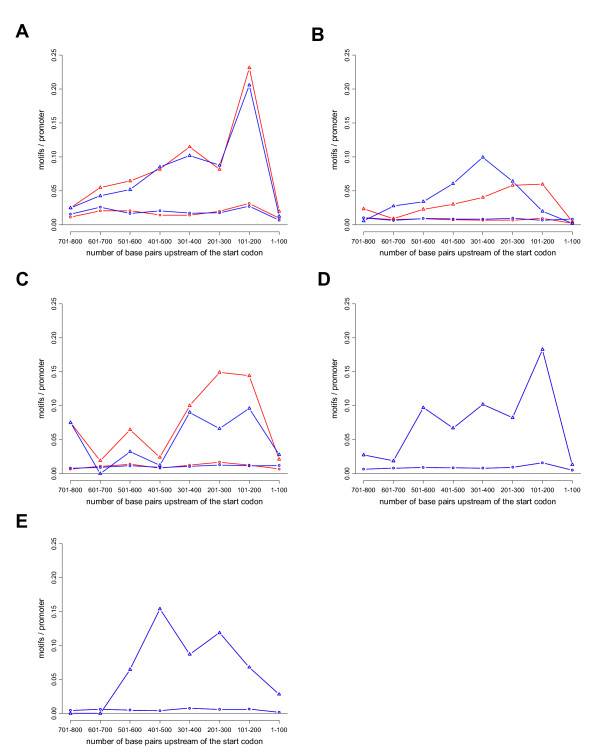
**Distribution of motifs in promoters bound by different TFs**. The *x *axis represents the distance to the start codon and the *y *axis the fraction of promoter sequences containing the motif of interest. The red and blue lines show the distribution of the two orientations of the motif. The two top lines (with triangles) show motif distribution in the set of promoters bound by the TF and the two lines at the bottom (with circles) show the distribution in the control set. The following motif-experiment pairs are shown: A) the Abf1 motif (cgtn{6}tga) in promoters bound by Abf1 on YPD, B) the Rap1 motif (ccrtaca) in promoters bound by Rap1 on YPD, C) the Gcn4 motif (gagtca) in promoters bound by Gcn4 on YPD, D) the Mbp1 motif in promoters bound by Mbp1 on YPD, E) the Gal4 motitf (cggn{11}ccg) in promoters bound by Gal4 on galactose. The corresponding p-values for location bias and orientation bias are shown in Table 2.

A few examples where motif context appeared to be important for DNA binding are shown in Figure 1, with the corresponding p-values given in Table 2. The first example is Abf1. We found a clear bias in the locations of the Abf1-binding motif in promoters that actually bind Abf1, with the most common location being 101–200 bp upstream of the start codon (Figure 1a). Abf1 is in fact one of the few cases where a location bias has been previously described [38]. Regulation by Rap1 is also known to be dependent on the locations and orientations of its binding motif, with a preference for positions 150–450 [22] or 100–600 [38]. Consistent with this, we found a significant location bias for DNA binding of Rap1 (Figure 1b). The majority of cases where we found a location bias associated with DNA binding are, however, new. Two such cases are Gcn4 (Figure 1c), where the majority of the motifs in promoters actually bound by Gcn4 are located 200–400 bp upstream of the start codon, and Mbp1 (Figure 1d), where motifs are preferentially found 100–200 bp upstream of the start codon. Gal4 (Figure 1e) is a third interesting example, since the preferential distance between Gal4-binding motifs and start codons is longer than in most other cases, with a peak at 401–500 bp.

**Table 2:** Significance of location and orientation bias for selected motif-experiment pairs

**Figure**	Description*	**Location p-val**	**Orientation p-val**	**Nr selected promoters**
1 a	ABF1_Lee reduce (cgtnnnnnntga) in ABF1_YPD	5.08E-10	1	547
1 b	RAP1_YPD (ccrtaca) in RAP1_YPD	4.19e-3	1	408
1 c	GCN4_Lee reduce (gagtca) in GCN4_YPD	1.94e-3	1	143
1 d	MBP1_lee reduce (acgcgt) in MBP1_YPD	8.27e-4	1	227
1 e	GAL4_lee reduce (cggnnnnnnnnnnnccg) in GAL4_RAFF	5.95e-3	1	71
4 a	PAC_ESR reduce (cgatgag) in group 4	2.89e-3	2.35e-1	114
4 b	rRPE_ESR reduce (aaaattt) in group 4	1.69e-12	1	114
4 c	RAP1_YPD (ccrtaca) in group 1	6.51e-12	3.08e-8	138
4 d	MBP1_lee reduce (acgcgt) in group 30	3.07e-14	1	52

* Should be read as: <motif name and source> (<motif sequence>) in <experiment name>.

**Figure 2:**
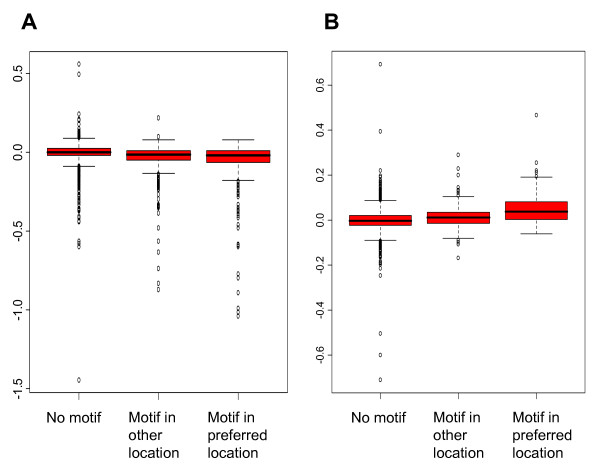
**Influence of motif location on gene expression in deletion strains**. A) Expression in the Gcn4 deletion strain compared to the wild type, for genes without the Gcn4 motif, with the motif outside the preferred location (200–400 bp upstream) and with the motif in the preferred position. B) Expression in Mbp1 deletion strain compared to the wild type, for genes without the Mbp1 motif, with the motif outside the preferred location (100–200 bp upstream) and with the motif in the preferred position.

To test if the observed bias in motif location within promoters that actually bind a given TF correlates with TF-specific effects on gene expression, we used available data on gene expression in different knockout strains [30]. We found such data for four of the TFs that showed a significant location bias: Cin5, Gcn4, Mbp1 and Swi4. For each of these TFs we compared expression of three sets of genes a) all genes without the motif in the promoter, b) all genes with the motif but not in the preferred location, and c) all genes where the promoter contains the motif in the preferred location. Gcn4 is an activator of genes that are induced in response to amino acid starvation, and as expected genes with the Gcn4-binding motif in their promoters have a reduced expression in the Gcn4 deletion strain. Notably, genes with a Gcn4 motif at 200–400 bp upstream of the start codon have a significantly lower (p = 9.4e-3) expression in the deletion strain than genes with the motif in other locations (Figure 2a). This shows that the location of the Gcn4-binding motif is important not only for Gcn4 binding, but also for Gcn4-dependent regulation *in vivo*. Mbp1 is a repressor involved in regulation of cell cycle progression, and as expected genes with the Mbp1-binding motif in their promoters have a higher level of expression in the Mbp1 deletion strain. Also in this case, we found that genes where the Mbp1 motif is found in a preferential location for DNA binding (101–200 bp upstream of the start codon) have a significantly higher (p = 8.4e-6) level of expression in the Mbp1 deletion strain than genes which have Mbp1 motifs elsewhere in their promoters (Figure 2b), suggesting that the location of the Mbp1 motif also is important for Mbp1-dependent repression. For Swi4 the results were inconclusive (p-value 0.095), and for Cin5 no expression differences were observed for different motif locations (data not shown).

**Figure 3:**
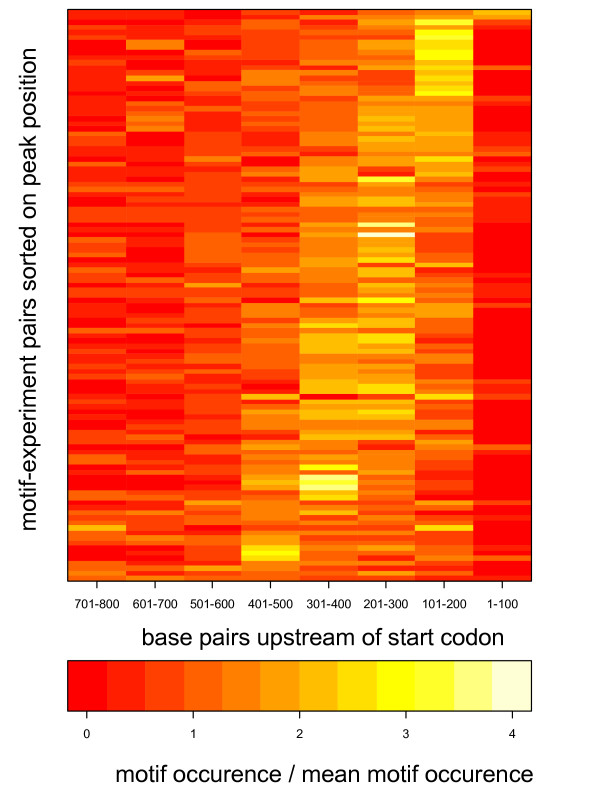
**Distribution of motifs along the promoters for different motif-experiment pairs**. The 113 motif-experiment pairs with a significant location bias were sorted according to which part of the promoters that contained most motifs. Colours indicate motif occurrence (normalized to the mean motif occurrence for each motif-experiment pair).

Several other cases of location bias were found, for TFs such as Gal4, Gcr1, Hap4, Hsf1, Mcm1, Pho4, Rcs1, Reb1, Skn7 and Ste12. To get an overview of the locations in the promoter regions that are preferred for DNA binding, we ordered all motif-experiment pairs with a significant location bias according to the location of the peak of the highest occurrence of each motif. The results are shown in Figure 3. Here, we find examples of peaks at different distances from the start codon: 101–200 bp (Abf1, Reb1), 201–400 (Rap1, Mcm1) and 401–500 bp (Gal4). For the majority of the motif-experiment pairs the peaks in motif occurrence were found between 101 and 400 bp upstream of the start codon, and none of the examples showed a preference for the first 100 bp upstream of the start codon, or for more distant positions, beyond 600 bp. This is consistent with the results in [19] and [23]. P-values on all motif-experiment pairs examined can be found in additional file 1: Table S1.

### Motif location and orientation is important for effects on gene expression

**Figure 4:**
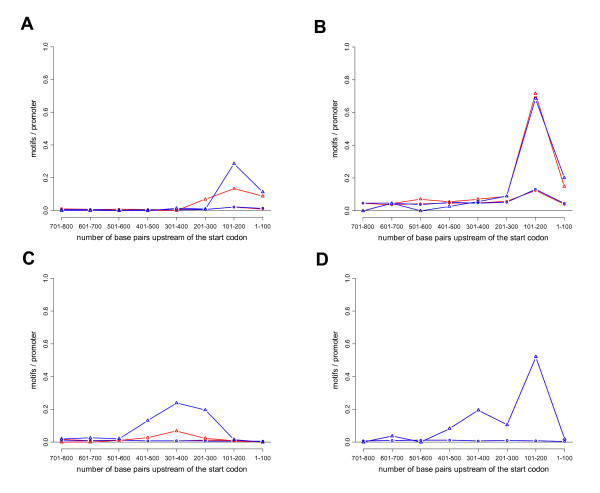
**Distribution of motifs in the promoters of sets of co-expressed genes**. The following motif-gene set pairs are shown: A) the PAC (cgatgag) motif in promoters of genes in group 4, B) the rRPE motif (aaaattt) in promoters of genes in group 4, C) the Rap1 motif (ccrtaca) in promoters of group 1, D) the Mbp1 motif in promoters of genes in group 30. The corresponding p-values for location bias and orientation bias are shown in Table 2.

A different question from the effects of motif position or orientation on TF binding is whether sets of co-expressed genes also show a bias for location or orientation for TF-binding motifs that are shared by these genes. It should be noted that for a given TF to regulate its target genes, it not only has to be able to bind to the DNA, but also has to interact correctly with other molecules, such as the basic transcription machinery and various co-factors. These interactions may introduce additional constraints on motif location or orientation. We therefore expected location or position bias for TF-binding motifs to be even more common among promoters of co-regulated genes than among promoters that simply bind a given TF. As shown in Table 1, this is indeed the case. Thus, out of the 23 motif-group pairs that we examined, we found that 57% (13 pairs) exhibit location bias and 22% (5 pairs) orientation bias (Table 1, third row). These numbers are higher than those associated with just DNA binding (see above). In particular, we note that orientation bias seems to be more common among co-expressed genes, as it was not seen when looking at just DNA binding. These results are in accordance with [24], where location and orientation bias were also frequently correlated with co-expression. Below we discuss some examples of sets of co-regulated genes that show position and/or orientation bias for TF-binding motifs (the corresponding p-values are shown in Table 2):

One group of co-expressed genes (number 4) has the PAC and rRPE motifs enriched in the promoters. Both motifs have a significant location bias, to positions 101–200 (Figures 4a and 4b). The location bias of the PAC and rRPE motifs has previously been reported in [12] and [4]. Another example is the Rap1 motif that is enriched in another set (number 30) of co-expressed genes. As shown in Figure 4c, Rap1 motifs in this set of genes are concentrated to 201–500 bp upstream of the start codon, and we also found a significant orientation bias. It is interesting to note that the constraints on the Rap1 motif are stronger in the promoters of the co-expressed genes than in promoters that bound Rap1 in ChIP-chip experiments (Figure 1b). These results suggest that Rap1-dependent gene expression imposes stronger constraints than just Rap1 binding on the positions and orientations of the Rap1 motif. The same tendency was seen for Mbp1 motif in set number 30 of co-expressed genes (Figure 4d). Thus, this motif is primarily located 101–200 bp upstream of the start codon, and the location bias is more pronounced than in promoters that just bind Mbp1 in the ChIP-chip experiment (Figure 1d).

In addition to the examples discussed above, location and/or orientation bias was found for the following TF binding motifs: Fkh1/2, Hap4, Msn2/4, Rpn4, and Yap1. The complete results can be found in additional file 2: Table S2.

### Preferred motif locations are negatively correlated with nucleosome occupancy

**Figure 5:**
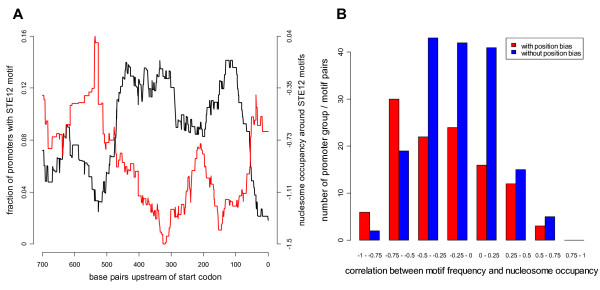
**Anti-correlation between motif frequency and nucleosome occupancy**. A) An example of anti-correlation between motif frequency and nucleosome occupancy. The black curve shows the distribution of Ste12 binding motifs within promoters bound by Ste12. The red curve shows the average nucleosome occupancy levels around these Ste12 sites. The correlation in this example is -0.79. B) Histogram over the correlation between motif frequency and nucleosome occupancy for all 280 motif-experiment pairs from the TF-DNA interaction data. Pairs with a significant location bias (in red) have significantly lower correlations (p-value 2.9e-3) than pairs without correlation bias (blue).

Since nucleosomes and TFs frequently compete for binding to DNA, nucleosome positions affect the DNA binding of many TFs. Furthermore, it has been shown that active TF binding sites are depleted of nucleosomes, as compared to inactive sites [27,28]. We therefore proceeded to use available nucleosome position data from yeast in an attempt to validate our results. Specifically, we expected motifs in preferred locations to be more likely to be biologically active than motifs in other locations, and thus also to be more likely to be depleted of nucleosomes than motifs in other locations. As expected, we found that nucleosome occupancy shows an inverse correlation with motif occurrence in promoters that bind a given TF. This is illustrated in Figure 5A for promoters that bind Ste12. When the entire set of data from the TF-DNA interaction studies [19,32] was examined, we found that instances with location bias for DNA binding show significantly (p-value 2.9e-3) higher anti-correlation between nucleosome occupancy and motif occurrence than instances without location bias (Fig 5b, for full results see additional file 3: Table S3). We conclude that motifs in preferred locations generally have less nucleosomes bound at or near them than motifs in other locations. In contrast, we did not see the same effect for motifs in promoters of co-expressed genes (p-value 0.16). We note, however, that much of the protein-DNA interaction data was obtained during exponential growth on YPD (Yeast Peptone Dextrose) as was the nucleosome occupancy data, while the expression data was obtained during several different conditions. Furthermore, there were fewer motif-group pairs in this case than for the DNA binding data, which makes this negative result harder to interpret.

## Discussion

It is becoming increasingly clear that the mere presence of a TF-binding motif in a promoter is not sufficient for correct gene regulation by that TF *in vivo*, but that the promoter context within which a motif is found also may have a significant effect. The short motifs recognized by TFs, typically six bp or less, are ubiquitously found in genomes, but only a small fraction of these motifs have been shown to be involved in gene regulation. Genome-wide location studies [18] have shown clear patterns of location bias in motifs bound by TFs *in vivo*. The study by Nguyen et al. [22] showed, for a few selected examples, that the same TF binding motif can have different effects on gene expression depending on the location and orientation of the motif. Tabach et al. [23] showed that promoters of genes sharing functional annotations in the human and the mouse are often enriched for motifs in a region close to the transcription start site. Moreover, the study by Elemento et al. [24] found that location and orientation bias was common among yeast motifs (but interestingly not *P. falciparum *motifs) in promoters of co-expressed genes. On the other hand, Yuan et al. [21] found that including information about motif context in their model did not improve predictions concerning gene expression. However, as pointed out by the authors themselves, this does not necessarily mean that motif context is biologically unimportant. The lack of predictive power when motif context was included in the model could be explained by increased model complexity, which makes training a general model more difficult. This is especially true for large scale models that intend to cover all the regulatory events in a cell, such as the one used in [21]. Thus, the question of how the promoter context influences the biological effects of TF-binding motifs is still largely unsolved. Our study presents the first genome-scale examination where both motif location and orientation is correlated with TF-DNA interactions and well as with co-expression data. For this, we have developed a new tool, ContextFinder. It is specifically aimed at finding and characterizing biologically significant differences in motif context on a genome-wide scale.

ContextFinder is based on a sound statistical framework (see Methods) and works with a wide range of data. ContextFinder does not require any parameter tuning, all that is required is one or several sequence motifs, a set of promoters that has been chosen for study, and a control set to which this set is compared. The set of promoters can be obtained from DNA biding data, expression data, or in some other way. The output of the method is the significances, in the form of p-values, for biases in motif location and orientation. Estimating the performance of ContextFinder is difficult since in general we cannot tell whether a given location or orientation bias is "true" or "false" in the sense that it reflects a biologically important preference that has been selected during evolution. Given that our statistical model is sound, we expect a false discovery rate of 5%. Thus, we expect the majority of the instances of location and orientation bias that are found by ContextFinder to be "true" positives. It is harder to estimate the number of false negatives, since there are a number of possible error sources. One comes from the pre-selection step where we only consider cases of motifs significantly enriched in a given set of genes. This means that we may remove some "true" positives from the subsequent analysis. Another source of error is the lack of a sufficient number of motif occurrences in order to obtain good statistics. For small sets of promoters, or for long and specific motifs, such scarcity of data can lead to "false" negatives. For these reasons, we expect our procedure to be rather conservative.

An overview of how common location and orientation bias is when our method is applied to sets of promoters chosen either from TF-DNA interaction data or from gene expression data is shown in Table 1. Although these numbers depend on the experimental details in each case, they can still provide an estimate of how important motif context is for DNA binding by TFs and their effects on gene expression, respectively. Our results suggest that motif location (but not motif orientation) frequently is important for DNA binding by TFs. Most TFs with location constraints seem to have a preference for motifs that are located 101–400 bp upstream of the coding sequence, which is close to the transcription start sites (located approximately 70 up upstream of the start codon). This may indicate that, for many TFs, interactions with the basal transcriptional machinery are required for stable binding to DNA. However, some TFs, such as Gal4, seem to prefer motifs further upstream.

Unlike the case with DNA binding, when we examined sets of co-expressed genes, we also found bias in the orientation of TF binding motifs. Location bias was also more common among promoters of co-expressed genes, than among promoters that simply share the fact that they bind the same TF. These results seem intuitive, since the activity of a TF in gene regulation involves not only its binding to DNA (which as we have seen above imposes constraints on motif location), but also interaction with other molecules and complexes such as the basal transcriptional machinery or co-factors: This may introduce additional constraints on the location and orientation of the motif.

It should be noted that by using ContextFinder on DNA binding data together with expression data it is possible to draw conclusions concerning the likely source(s) of any context biases found for a given motif. For example, if a motif context is important already for DNA binding, and does not change in the expression data, it is likely that the motif context is required for stable DNA binding. On the other hand, in cases where motif context is important only for gene expression, but not for DNA binding of a TF, we can infer that the processes subsequent to DNA binding by the TF that require a specific motif context. Finally, there may exist cases where some context bias is seen in DNA binding, with further constraints apparently being imposed for the TF to be active in gene regulation. Rap1 and Mbp1 are examples of this.

We have validated our results against two independent data sources: The first is global gene expression data from yeast deletion strains that lack individual TFs [30]. Our results show that there is a significant difference in the effects of these TF deletions on the expression of genes containing binding motifs for the given TF in either the preferred location or in other locations. We have further shown that there is an anti-correlation between motif occurrence and nucleosome occupancy, so that TF-binding motifs in preferred locations are depleted of nucleosomes as compared to motifs in other locations. Similar results were obtained in [26] for a few examples (Abf1, Reb1 and Mbp1) where the motifs clustered to a region within 80–100 of the transcriptions start site. Since we used additional data to distinguish between biologically active and cryptic motifs, we found many more cases of anti-correlation between nucleosome occupancy and the locations of motifs (see additional file 3: Table 3), also for motifs that are preferentially located further upstream than 100 bp. We interpret the anti-correlation between motif occurrence and nucleosome occupancy, as well as the observed differences in gene expression that correlate with the locations or orientations of motifs, as evidence that motif context in these cases has biological relevance.

There are several possible mechanisms by which motif context could affect DNA binding or activity of individual TFs. Since all TFs studied here tend to bind within 600 bp upstream of the start codon (and most within 400 bp), interactions with the basic pol II transcription machinery are likely to be important. The cases of orientation bias that we found for sets of co-expressed genes could also be due to interactions with the pol II complex or with co-factors, which require the TF to be positioned in a certain way. It is also possible that the induced changes in DNA conformation that are needed for gene regulation, such as DNA bending or unwinding, may impose constraints on the locations and orientations of TF-binding sites. One obvious case is binding of TBP to the TATA-box, a motif which shows strict orientation bias. As for the effects of nucleosome positioning, the region immediately (1–200 bp) upstream of the transcription start site is usually depleted of nucleosomes [26,28]. Since this region is also enriched for many TF binding sites (*e. g. *Abf1, Reb1, Mbp1) it may be the case that the ability to bind DNA, which is determined by nucleosome positioning, is the reason why motif context is important for these TFs. However, this does not apply to other TFs, such as Gal4, Rap1 and Swi4, whose binding sites are found further upstream in regions with high nucleosome occupancy. Thus, it is likely that several different mechanisms contribute to the observed biases in motif location and orientation.

## Conclusion

In this paper we have presented a new method to identify constraints on motif location and orientation, that may be imposed by the need for stable DNA binding and/or the regulatory functions of transcription factors. Our method is based on a generalized linear model, and outputs p-values describing the significances of any biases in motif locations and orientations.

We used this method to analyse 303 cases of motifs enriched in experimentally selected groups of yeast promoters. Bias in motif location was found to be common for motifs that were enriched in promoters identified as being bound by a specific TF in TF-DNA interaction experiments, whereas bias in both location and orientation was found for motifs enriched in promoters of co-expressed genes. Furthermore, motifs in preferred locations were depleted of nucleosomes, compared to motifs in other locations. These results suggest that motif context is likely to be an important mechanism responsible for TF specificity in gene regulation.

We conclude that when using motif information to predict gene regulatory relationships, information about motif locations and orientations may have to be considered in addition to the mere presence or absence of a motif. We provide the first generally available method to find and characterize biases in motif context, that may easily be accessed though a web interface.

## Methods

### Modelling binding site occurrences

In order to study if the distribution of a motif differs significantly between a selected set of promoters and a control set, we modelled the (log) probability of finding a motif as a function of the distance to the start codon, the orientation of the motif and which set the promoter belongs to. In more detail, we modelled the number of occurrences of a motif *y*_*g*, *o*, *l *_as dependent on location *l *(1–100, 101–200, 201–300, 301–400, 401–500, 501–600, 601–700 or 701–800 bp upstream of the start codon), orientation *o *(+ or -) and set of promoters *g *(selected or control). Since the promoter sequences are of variable length, the number *n*_*g*, *l *_of available promoters at the given location was also included in the model. To detect any bias in location and orientation of motifs, a generalized linear model with a Poisson distribution [39], was fitted to the data:

$$\log\left(\frac{y_{g,o,l}}{n_{g,l}}\right)=\mu +\alpha_{g}+\beta_{o}+\chi_{l}+{\left(\alpha \beta \right)}_{g,o}+{\left(\alpha \chi \right)}_{g,l}+{\left(\beta \chi \right)}_{o,l}$$

Here *y*_*g*, *o*, *l *_is the number of promoters containing the motif, *n*_*g*, *l *_is the number of available promoters, *μ *is the intersect, *α*_*g *_is the effect of promoters belonging to the group *g*, *β*_*o *_is the effect of motif orientation *o*, and *χ*_*l *_is the effect of the location *l*. The model also contains interaction effects: (*αβ*)_*g*, *o *_between group and orientation, (*αχ*)_*g*, *l *_between group and location and (*βχ*)_*o*, *l *_between orientation and location. After the data has been fitted to the model, the null hypothesis that each coefficient is equal to zero is tested, using the residual deviance. For each coefficient, the residual deviance follows a *χ*^2 ^distribution (with the same number of degrees of freedom as the coefficient), which enables us to compute a p-value [39]. The coefficients of interest to us are (*αβ*)_*g*, *o *_(*orientation bias*, indicating differences in orientation between the two sets of promoters,) and (*αχ*)_*g*, *l *_(*location bias*, indicating differences in location). These coefficients were considered significant if the corresponding p-value was below a given threshold. Since many pairs of motifs and promoter groups were considered, the p-values were adjusted for multiple hypothesis testing [40]. The threshold used in our analysis corresponds to a false discovery ratio of 0.05.

As a test of whether it was reasonable to assume a Poisson distribution, we checked for over-dispersion. Dispersion values were computed by dividing the residual deviance from the full model with the degrees of freedom [39]. In ~95% of the cases the dispersion was below 2. For the 5% cases with higher dispersion, the p-values of the coefficients were adjusted accordingly [41]. This procedure did not change the overall results significantly. The program ContextFinder implements this method (in R). A web interface to the program is available at [36] and the source code is available upon request (and in the process of submission to BioConductor [42]).

### Data

All available yeast promoter regions were retrieved from the RSAT database [43]. The promoter regions ranged from the start codon and 800 bp upstream or until the next ORF was reached, resulting in sequences of variable length. Since the distance between start codon and transcription start site is usually fixed (at around 70 bp) in *S. cerevisiae *[33-35], we used the start codon, which is easier to locate, instead of the transcription start site. This is not likely to have a major effect on the results, particularly since we use bins of 100 bp in our analysis. As the set of motifs to analyze, we used a list of 150 putative TF binding sites (represented as IUPAC strings) from [37], along with a few additional motifs, such as the PDS element [44].

To identify promoters that are bound by a specific TF, ChIP-chip data from 350 experiments (using different TFs and growth conditions) from Lee et al. [32] and Harbison et al. [19] were used. For each experiment, all promoters with p-values below 0.01 were considered to be bound by the TF, and all other promoters were used as the control set.

To examine promoters of co-regulated genes, the grouping of genes from [12] was used. The genes in that study were clustered on expression data from two studies: response to different types of environmental stress [29] and progression through the cell cycle [31]. This resulted in 49 sets of genes. For each set, the promoters in all other sets were used as the control set.

The next step was to find motif-experiment pairs that could be used for further analysis, *i.e. *where the motif was significantly enriched among the selected promoters. Motif enrichment was tested using a one-sided hypergeometrical test on the number of selected promoters with and without the motif, compared to the number of control promoters with and without the motif. Since the number of the tested motif-experiment pairs was large, the threshold for motif enrichment was set rather strict, to 1e-8. This resulted in 292 motif-experiment pairs from the DNA binding data, and 26 motif-gene set pairs from the gene expression data. These pairs were then tested for context dependence.

When groups of promoters are analyzed together for motif context there is a risk that the results will be misleading if the promoters are highly conserved. Thus, if there is high sequence conservation among a group of promoters, the location and orientation bias that we may find will not be informative, since such bias would be detected for almost any sequence present in the promoters. To handle this, we checked for conservation for each analyzed motif-experiment pair that had a significant location or orientation bias, by aligning all selected promoters containing the motif of interest. The alignment was done in ClustalW (implemented in the R-library *dna*, [45]), using default parameters (gap opening penalty 15 and gap extension penalty 6). Twelve cases from the DNA binding data and three from the expression data with highly conserved promoters were removed from the subsequent analysis. See the additional file 1: Table S1 and additional file 2: Table S2.

### Validation against other datasets

To check whether motif position had any effect on gene expression, microarray data from yeast deletion strains [30] was used. T-tests were used to compare expression of genes whose promoters contained the motif of interest in the preferred location, against expression of genes whose promoters contained the motif in some other location.

The relationship between nucleosome occupancy and motif frequency was examined in the following way: For each motif-experiment pair, a sliding window procedure (window size *w *= 100 bp) was used to count the number of motifs in the selected set of promoters. For all promoter positions *i *between 1 and 800-*w*, let *M*_*i *_be the set of all motifs between *i *and *i+w *bp upstream of the start codon. The average nucleosome occupancy around the motifs across the promoters was defined as:

$$n_{i}=\frac{1}{\left|M_{i}\right|}\sum_{m\in M_{i}}nuc\left(m\right)$$

where *nuc(m) *is the average nucleosome occupancy (taken from [26]) of 50 bp around motif *m*. The motif occurrence was given by:

$$k_{i}=\frac{\left|M_{i}\right|}{p_{i}}$$

where, *p*_*i *_is the number of available promoters at *i *base pairs upstream of the start codon and *M*_*i *_is defined as above. We then computed the correlation between *n *and *k *for each motif-experiment pair. The correlations for cases with and without location bias were then compared using Wilcoxon's rank-sum-test.

## Authors' contributions

JOW conceived of the study, designed the methods, carried out the analysis and wrote a draft manuscript. JOW and FX wrote the source code. HR and JK revised the draft manuscript and led the project. All authors read and approved the final manuscript.

## Supplementary Material

Additional file 1 **Analysis of motif context in promoters bound by transcription factors.** The data provided show the results of Context finder for all motif – experiment pairs from the DNA binding data. For each such pair the following values are provided: significance of orientation bias (p-value), significance of location bias (p-value), dispersion and the number of selected promoters. The results in this file are summarized in the first row of Table 1. Click here for file

Additional file 2 **Analysis of motif context in promoters of co-expressed genes.** The data provided show the results of Context finder for all motif – experiment pairs from the co-expression data. For each such pair the following values are provided: significance of orientation bias (p-value), significance of location bias (p-value), dispersion and the number of selected promoters. The results in this file are summarized in the third row of Table 1. Click here for file

Additional file 3 **Correlation between motif frequency and nucleosome occupancy.** The data provided show the correlation between motif frequency and nucleosome occupancy for all motif – experiment pairs from the DNA binding data. The results in this file are summarized in Figure 5b. Click here for file
